# Demonstration of extracellular peptidylarginine deiminase (PAD) activity in synovial fluid of patients with rheumatoid arthritis using a novel assay for citrullination of fibrinogen

**DOI:** 10.1186/s13075-014-0498-9

**Published:** 2014-12-05

**Authors:** Dres Damgaard, Ladislav Senolt, Michael Friberg Nielsen, Ger J Pruijn, Claus H Nielsen

**Affiliations:** Institute for Inflammation Research, Department of Infectious Diseases and Rheumatology, Copenhagen University Hospital, Rigshospitalet, Section 7521, Blegdamsvej 9, DK-2100 Copenhagen, Denmark; Institute of Rheumatology and Clinic of Rheumatology, 1st Faculty of Medicine, Charles University, Na Slupi 4, 128 50 Prague 2, Czech Republic; Department of Biomolecular Chemistry, Institute for Molecules and Materials, Radboud Institute for Molecular Life Sciences and Netherlands Proteomics Centre, Radboud University Nijmegen, P.O. Box 9101, NL-6500 HB Nijmegen, The Netherlands

## Abstract

**Introduction:**

Members of the peptidylarginine deiminase (PAD) family catalyse the posttranslational conversion of peptidylarginine to peptidylcitrulline. Citrullination of proteins is well described in rheumatoid arthritis (RA), and hypercitrullination of proteins may be related to inflammation in general. PAD activity has been demonstrated in various cell lysates, but so far not in synovial fluid. We aimed to develop an assay for detection of PAD activity, if any, in synovial fluid from RA patients.

**Methods:**

An enzyme-linked immunosorbent assay using human fibrinogen as the immobilized substrate for citrullination and anti-citrullinated fibrinogen antibody as the detecting agent were used for measurement of PAD activity in synovial fluid samples from five RA patients. The concentrations of PAD2 and calcium were also determined.

**Results:**

Approximately 150 times lower levels of recombinant human PAD2 (rhPAD2) than of rhPAD4 were required for citrullination of fibrinogen. PAD activity was detected in four of five synovial fluid samples from RA patients and correlated with PAD2 concentrations in the samples (*r* = 0.98, *P* = 0.003). The calcium requirement for half-maximal activities of PAD2 and PAD4 were found in a range from 0.35 to 1.85 mM, and synovial fluid was found to contain sufficient calcium levels for the citrullination process to occur.

**Conclusions:**

We present an assay with high specificity for PAD2 activity and show that citrullination of fibrinogen can occur in cell-free synovial fluid from RA patients.

## Introduction

Posttranslational modifications, such as citrullination, methylation or glycosylation, are common alterations that modify protein structure and possibly stability, functionality and antigenicity of proteins. The peptidylarginine deiminases (PADs) are a family of enzymes capable of converting arginine residues in (poly)peptides to citrullines, a process known as *citrullination*. Five PAD isoforms (PADs 1 to 4 and PAD6), with differential cellular and tissue distribution, have been described in humans [1]. Citrullination regulates homeostatic processes such as keratinocyte differentiation [2] and maintenance of myelin sheath insulation [3]. Moreover, citrullination is involved in the innate immune response—that is, by regulation of chemokine activity [4]—and by formation of neutrophil extracellular traps [5]. Citrullination has been linked to the pathogenesis of an increasing number of diseases (reviewed in [6]), with rheumatoid arthritis (RA) being the most intensively explored example [7]. In RA, PADs play a crucial role in the generation of the citrullinated proteins targeted by anti-citrullinated protein antibodies (ACPAs) [7] and citrullinated peptides recognized by autoreactive T cells [8]. Thus, a major proportion of RA patients carry human leucocyte antigen molecules containing the “shared epitope” motif [9] capable of binding citrullinated self-peptides [10]. The different isoforms of PAD have different substrate specificities [11] and may thus generate different citrullinated peptides for recognition by self-reactive T cells. Increased levels of PADs and hypercitrullination of proteins have been linked to other chronic inflammatory diseases, including neurodegenerative conditions such as multiple sclerosis [12] and Alzheimer’s disease [13].

Activity of PADs has been demonstrated in various cell lysates, such as muscle, ovary and spinal cord [14]. The presence of PADs has been shown in synovial fluid from RA patients as well as from patients with osteoarthritis (OA) [15]. Although numerous citrullinated proteins have been described in synovial fluid of RA patients [16], it has not, to our knowledge, been shown that the conditions necessary for PADs to be active, including an appropriate calcium concentration, are met in synovial fluid. Binding of calcium to PADs alters the conformation of the catalytic site and thereby PADs’ interaction with various substrates [17]. Depending on the method of detection, half-maximal PAD activity has been reported at calcium concentrations ranging from 40 μM to 3.3 mM [14,18,19]. The low intracellular calcium levels of resting cells indicate that intracellular citrullination occurs only after cell stimulation or after membrane disintegration as a result of cell death [20]. Both intracellular proteins (for example, vimentin) and extracellular proteins (for example, fibrinogen) are targets for ACPAs in RA [21,22], and the citrullinated forms of vimentin and fibrinogen and fibrin have been shown to enhance a proinflammatory immune response in ACPA-positive patients [23–25]. It is unclear which citrullinated antigens are more important in the pathogenesis.

Several methods for measurement of PAD activity have been described. These assays usually measure conversion of arginine to citrulline in synthetic peptides by means of colorimetry [26], high-performance liquid chromatography, fluorometry [27], fluorescence quenching [28], spectrophotometry [29] and antibody-based detection [14] and differ with respect to sensitivity and applicability for biological samples [30]. None of them are capable of discriminating between citrullination caused by the different PAD isotypes, however.

Different substrate specificities have been demonstrated for PAD2 and PAD4 [11,31], with PAD4 showing a more restricted pattern than PAD2. In the present study, we exploited these differences to develop an assay, measuring citrullination of fibrinogen, with almost complete specificity for PAD2 activity. This assay provides new insight into the calcium requirement of PAD2 and PAD4, and we used it to determine the catalytic activity of PADs in synovial fluid samples.

## Methods

### Collection of synovial fluid from rheumatoid arthritis patients

Synovial fluid samples were obtained during joint aspiration for therapeutic reasons from five patients with RA (four women). The patients fulfilled the American College of Rheumatology criteria for the diagnosis of RA [32] and gave us their written informed consent for participation. The mean age (range) of the patients was 58.6 (45 to 69) years. Four patients were positive for ACPAs. All samples were stored at −80°C until use. Before analysis, synovial fluid samples were treated with hyaluronidase (Hylase Dessau; Riemser Arzneimittel, Greifswald, Germany) at 37°C for 30 minutes and centrifuged at 3,500 rpm for 10 minutes. The study was approved by the local ethics committee of the Institute of Rheumatology, Charles University Prague, Czech Republic.

### Quantification of Ca^2+^ concentration in synovial fluids

Synovial fluid calcium concentrations were determined using a colorimetric assay based on formation of a chromogenic complex between free calcium and *o*-cresolphthalein (Abcam, Cambridge, UK). Absorbance was measured at 575 nm using the SPECTROstar Nano microplate reader (BMG Labtech, Ortenberg, Germany).

### Quantification of peptidylarginine deiminase 2 concentration in synovial fluids

The PAD2 content in synovial fluid samples was determined in a previously described assay [33]. In brief, enzyme-linked immunosorbent assay (ELISA) plates were coated with anti-PAD2 monoclonal antibody (mAb) *DN2* (1 μg/ml) overnight at 4°C. Synovial fluid samples were diluted twofold from 1:10 to 1:80 in dilution buffer (phosphate-buffered saline (PBS), 0.5% Tween 20, 2% bovine serum (Sigma, St Louis, MO, USA), 20 μg/ml Mouse Immunoglobulin G Isotype Control (Novus Biologicals, Cambridge, UK), pH 7.4) and incubated for 2 hours at room temperature. Biotinylated anti-PAD2 mAb *DN6* (1 μg/ml) was added, followed by incubation with streptavidin-conjugated horseradish peroxidise (HRP; Invitrogen, Carlsbad, CA, USA) and development with *o*-phenylenediamine substrate (Kem-En-Tec Diagnostics, Taastrup, Denmark). All standards and samples were measured in duplicates. Absolute PAD2 concentrations were calculated by regression analysis for the standard curve using four-parameter logistic curve-fitting by means of MARS software (BMG Labtech).

### Peptidylarginine deiminases

Recombinant human PAD2 (rhPAD2) and rhPAD4 were produced, purified and defined by means of mass concentration, as described previously [34]. Commercially available rhPAD enzymes ModiQuest (MQ) rhPAD2 (MQ21.101; 4.88 mU/ml) and MQ rhPAD4 (MQ21.102; 58.3 mU/ml) were all purchased from ModiQuest, Oss, Netherlands.

### Antibodies

Monoclonal mouse anti-citrullinated fibrinogen (anti-cFib) (clone 20B2) and human anti-cFib (clone 1 F11) were purchased from ModiQuest (MQ13.102 and MQR2.101, respectively). HRP-conjugated polyclonal rabbit anti-mouse antibodies were obtained from Dako (Glostrup, Denmark).

### *In vitro* citrullination of fibrinogen

Fibrinogen (Calbiochem, Darmstadt, Germany) was incubated at a final concentration of 1.0 mg/ml with rhPAD2 (60 ng/ml) raised in-house in citrullination buffer (100 mM Tris-HCl, 10 mM CaCl_2_, 1 mM dithiothreitol (DTT), pH 7.5) overnight at 37°C. Mobility changes of citrullinated fibrinogen were confirmed with SDS-PAGE.

### Enzyme-linked immunosorbent assay activity

Nunc MaxiSorp ELISA plates (Roskilde, Denmark) were coated overnight at 4°C with 100 μl of fibrinogen (1.0 μg/ml) in coating buffer (30 mM Na_2_CO_3_, 70 mM NaHCO_3_, pH 9.6). Wells were washed thrice and blocked in washing buffer A (Tris-buffered saline, 0.05% Tween 20, pH 7.4) for 20 minutes at room temperature. In general, rhPADs were diluted in citrullination buffer and incubated for 3 hours at room temperature for determination of activity and calcium dependency. Synovial fluid samples were applied diluted 1:3 in various buffers (Tris-HCl, 1 mM DTT with or without 10 mM CaCl_2_ or ethylenediaminetetraacetic acid (EDTA)). Following three washes in washing buffer B (PBS, 0.05% Tween 20, pH 7.4), the wells were incubated for 90 minutes at room temperature with 100 μl of murine anti-cFib antibody in washing buffer B. After three further washes, the wells were incubated with 100 μl of HRP-conjugated polyclonal rabbit anti-mouse immunoglobulin antibodies (Dako) diluted 1:1,000 in washing buffer B. Finally, the plates were washed thrice in washing buffer B and incubated with 0.4 mg/ml *o*-phenylenediamine in developing buffer (35 mM citric acid, 65 mM Na_2_PO_4_, pH 5.0). After 10 minutes, the colour reaction was stopped with 1.0 M H_2_SO_4_, and optical density (OD) was measured at 490 to 650 nm using the SPECTROstar Nano microplate reader (BMG Labtech). Data were processed using MARS software (BMG Labtech). This procedure was modified as indicated in the Results section.

### Statistical analysis

All experiments are shown as means and ranges of duplicate measurements. Pearson’s correlation (*r*) coefficient and levels of significance were determined using GraphPad Prism 5.0 software (GraphPad Software, La Jolla, CA, USA).

## Results

### Assay for detection of citrullinated human fibrinogen

**Figure 1:**
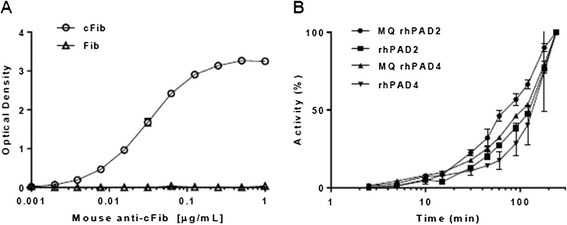
***In situ*** **citrullination of human fibrinogen. (A)** Fibrinogen (Fib) or citrullinated fibrinogen (cFib) was immobilized to enzyme-linked immunosorbent assay (ELISA) plates at a concentration of 1 μg/ml. Increasing concentrations of the anti-cFib monoclonal antibody (mAb; clone 20B2) were added, followed by addition of enzyme-conjugated rabbit anti-mouse immunoglobulin antibodies and *o*-phenylenediamine substrate. All data points represent means and ranges of duplicate measurements of optical density at 490 nm. **(B)** ELISA plates were coated with 1.0 μg/ml human fibrinogen and incubated with citrullination buffer including recombinant human peptidylarginine deiminase (rhPAD) enzyme, ModiQuest (MQ) PAD2 (8 mU/ml), rhPAD2 (42 ng/ml), MQPAD4 (49 mU/ml) or rhPAD4 (1,500 ng/ml). Shown is the activity as a percentage of the maximal activity obtained for each enzyme, corresponding to the activity at 4 hours. Anti-cFib mAb was used at a concentration of 0.5 μg/ml, as determined from the graph shown in (A). Symbols and error bars represent means and ranges of duplicate measurements.

We developed an assay for citrullination of immobilized human fibrinogen using a mAb specific for cFib as the detecting antibody (Figure 1A). Immobilized human fibrinogen was incubated with different rhPADs for various periods. Citrullination of fibrinogen was detectable as early as after 2.5 minutes, and the highest signals, without reaching a plateau, were observed after 4 hours of incubation (Figure 1B). A substantial degree of citrullination occurred after 3 hours of incubation, which was selected for subsequent studies.

### Comparison between enzymatic activities of peptidylarginine deiminases 2 and 4

**Figure 2:**
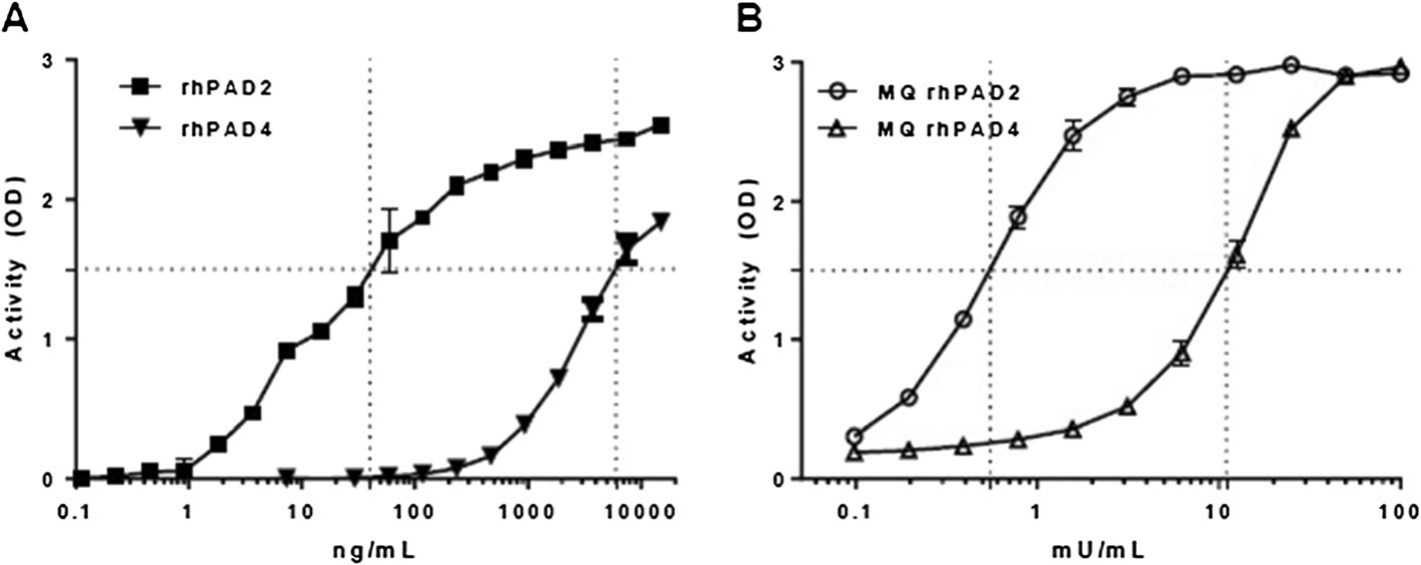
**Comparison between enzymatic activities of peptidylarginine deiminases 2 and 4.** Enzyme-linked immunosorbent assay plates were coated with 1.0 μg/ml human fibrinogen. **(A)** Recombinant human peptidylarginine deiminase 2 (rhPAD2) or rhPAD4 raised in-house were used for citrullination at mass concentrations ranging from 0.1 ng/ml to 15 μg/ml. **(B)** Commercially available ModiQuest (MQ) PAD2 or MQPAD4 enzymes were used in units ranging from 0.1 mU/ml to 100 mU/ml. Following a 3-hour citrullination period, the anti-cFib mAb (0.5 μg/ml) was used for detection of citrullinated fibrinogen. Duplicate measurements of optical density (OD) at 490 nm are shown as mean and range.

The assay proved markedly more sensitive to PAD2 activity than PAD4 activity, in that approximately 150 times more in-house rhPAD4 was required to achieve an extent of fibrinogen citrullination comparable to that catalysed by in-house rhPAD2 (Figure 2A). The lower limit of detection (LOD), defined as background plus 3 times standard deviation (SD), was 0.46 ng/ml for rhPAD2 and 234 ng/ml for rhPAD4. A similar pattern was observed for the recombinant enzymes obtained from ModiQuest, where ~20 times more units of MQPAD4 than of MQPAD2 were needed for similar levels of Fib citrullination (Figure 2B). The LOD for MQ rhPAD2 was 0.08 mU/ml and 1.25 mU/ml for MQ rhPAD4. Using a different antibody for detection, that is human anti-cFib antibody (clone 1 F11), we obtained similar preference for PAD2-mediated citrullination (data not shown).

### Calcium dependency of peptidylarginine deiminases 2 and 4

**Figure 3:**
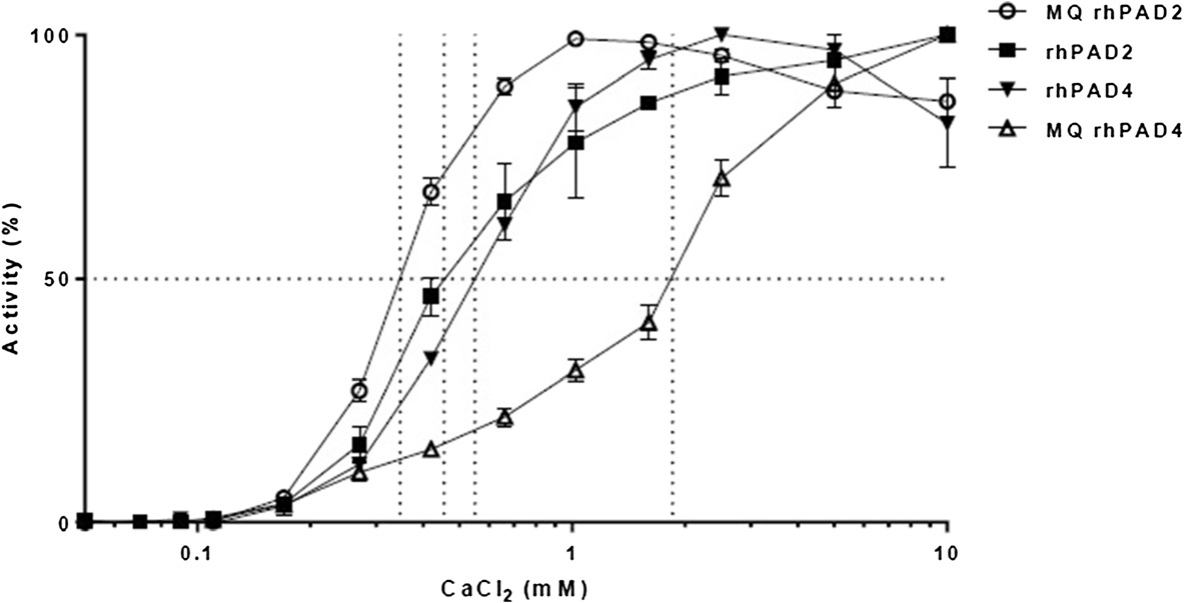
**Calcium dependency of peptidylarginine deiminases.** Enzyme-linked immunosorbent assay plates were coated with 1.0 μg/ml human fibrinogen and incubated with four different recombinant human peptidylarginine deiminase (rhPAD) preparations: ModiQuest (MQ) PAD2 (8 mU/ml), rhPAD2 (42 ng/ml), rhPAD4 (1,500 ng/ml) or MQPAD4 (49 mU/ml). Dithiothreitol was added at a concentration of 1.0 mM, and CaCl_2_ was added at concentrations ranging from 50 μM to 10 mM. Following 3 hours of incubation, citrullination was measured using mouse anti-cFib monoclonal antibody (0.5 μg/ml). Shown is the activity as a percentage of maximal activity for each enzyme. Symbols and error bars represent means and ranges of duplicate measurements.

The enzymatic properties of four different PAD preparations were tested at calcium concentrations ranging from 50 μM to 10 mM (Figure 3). No activity of any of the enzymes was observed below 110 μM calcium. Maximal activities were observed in the range from around 1.5 to 10 mM CaCl_2_. MQ rhPAD2, in-house rhPAD2 and in-house rhPAD4 showed similar requirements for calcium. The half-maximal activities of these enzymes were observed at CaCl_2_ concentrations of 0.35 mM, 0.46 mM and 0.55 mM, respectively. MQ rhPAD4 required markedly higher calcium concentrations for citrullination, showing half-maximal activity at 1.85 mM CaCl_2_.

### Peptidylarginine deiminase activity can be determined in synovial fluid from patients with rheumatoid arthritis

**Figure 4:**
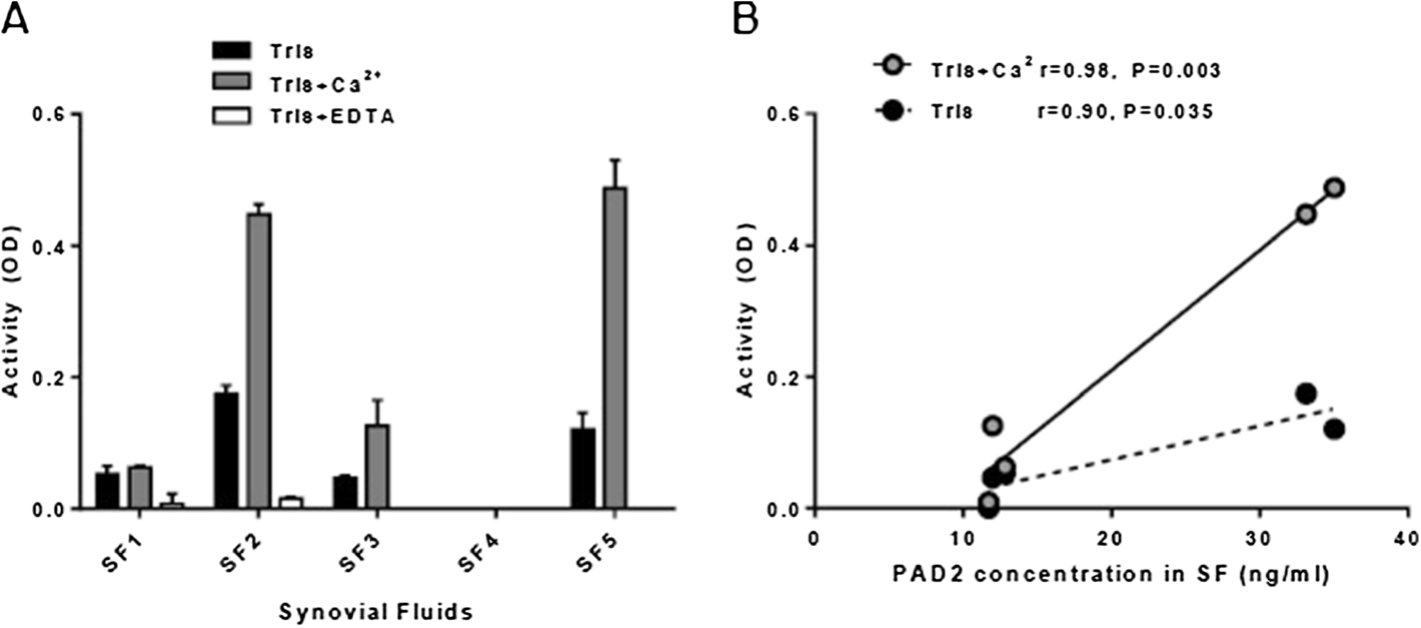
**Peptidylarginine deiminases activity in synovial fluid samples from rheumatoid arthritis patients. (A)** Synovial fluid (SF) samples from five rheumatoid arthritis patients were applied on enzyme-linked immunosorbent assay plates coated with fibrinogen (1 μg/ml) after 1:3 dilution into Tris buffer with 1 mM dithiothreitol (DTT; black columns), Tris buffer containing 1 mM DTT and 10 mM CaCl_2_ (grey columns) or Tris buffer containing 1 mM DTT and 10 mM ethylenediaminetetraacetic acid (EDTA; white columns). Following a 3-hour incubation period, the monoclonal mouse anti-citrullinated fibrinogen (anti-cFib) (0.5 μg/ml) was used for detection of citrullinated fibrinogen. Peptidylarginine deiminase (PAD) activity is presented as optical density (OD) at 490 nm of duplicated measurements. **(B)** Association between PAD2 concentration and PAD activity in the five SF samples diluted 1:3 in citrullination buffer containing 10 mM CaCl_2_ (grey circles, solid line) or no additive calcium (black circles, dotted line). Pearson’s correlation coefficients (*r*) and levels of significance are shown.

Synovial fluid samples from five patients with RA (SF1 to SF5) were tested for PAD activity. The calcium content in the samples ranged from 1.5 to 2.5 mM (data not shown), which was well within the range for PAD to be active, as shown above. Four of the five samples were capable of citrullinating fibrinogen after 1:3 dilution in Tris buffer, yielding a final calcium concentration of 0.5 to 0.83 mM (black columns in Figure 4A). Optimizing the enzymatic properties by addition of 10 mM CaCl_2_ to the buffer markedly increased the activity in three samples (SF2, SF3 and SF5) and to a minor extent in one sample (SF1). Addition of the calcium chelator EDTA eliminated activity in all samples (grey and white columns in Figure 4A, respectively).

The five samples all contained soluble PAD2, at concentrations ranging from 12 ng/ml to 35 ng/ml (*x*-axis in Figure 4B), and the PAD activity of the samples correlated with the PAD2 content (Figure 4B).

## Discussion

The presence of PAD2 and PAD4 have been demonstrated in synovium from RA and OA patients [15], and multiple citrullinated proteins are known to be present in synovial fluid from RA patients [16]. It has not been established, however, if the requirements for the two isoforms to be active are met in the synovium for citrullination to take place *in situ*. Alternatively, citrullinated proteins may be translocated to synovial fluid from other sites. Insight into the absolute and relative activities of different PAD isoforms in synovial fluid in various types of joint inflammation may lead to a better understanding of the pathophysiology of the diseases and may be diagnostically useful.

The assay for citrullination of human fibrinogen presented here showed a strong preference for detection of PAD2 activity compared to PAD4 activity. We modified an already existing antibody-based ELISA [14] by changing the enzyme substrate from arginine-rich peptides to full-length fibrinogen. Citrullinated fibrinogen is a well-established autoantigen in RA [22], with approximately two-thirds of RA patients having autoantibodies against this modified self-antigen [35]. It is well established that PAD2 and PAD4 citrullinate fibrinogen to different degrees and target different arginine residues [11,31]. The detecting antibody used in the present study (clone 20B2) apparently recognizes an epitope on fibrinogen that is preferentially citrullinated by PAD2. Therefore, it cannot be firmly concluded that PAD2 is more potent than PAD4.

The main finding of our study is that PAD activity was present in four of five synovial fluid samples from RA patients. In accordance with the PAD2 specificity of the assay, the PAD activity of the samples correlated with their PAD2 content. This finding does not exclude PAD4 activity in synovial fluid, however. Pure synovial fluid could not be applied to the assay, owing to the high viscosity of the samples, so it was necessary to apply a threefold dilution of the samples. The calcium concentration of the samples fell below the 1.5 mM required for optimal activity. Therefore, the activity observed after dilution in buffer containing 10 mM calcium for optimal activity presumably more closely reflects the activity of untreated synovial fluid.

We found a pronounced calcium dependency of PAD2 and PAD4, which both required calcium concentrations above 100 μM to be active. Some investigators have demonstrated half-maximal activity of rabbit PAD2 and rhPAD4 around 40 to 80 μM [14,18], whereas others have found roughly tenfold higher concentrations (200 to 750 μM) [11,29,36], in agreement with our findings. In a recent study, investigators found half-maximal activity of human PAD4 to be as high as 3.3 mM [19]. Use of different substrates in various assays or different enzyme purification procedures may account at least in part for these differences. Presumably, intracellular calcium concentrations above 100 μM can be achieved only in situations of pronounced membrane disruption after serious cell damage or death, and not even during apoptosis [37,38]. Factors other than calcium may enhance the catalytic properties of PADs, rendering intranuclear and/or intracellular activity possible. Thus, ATP induces intracellular, PAD2-mediated citrullination in mast cell cultures and facilitates release of citrullinated proteins and PAD2 itself [20].

## Conclusions

We have developed an assay to measure activity of PADs using a RA autoantigen—fibrinogen. The assay is highly specific for PAD2 activity, and we demonstrate catalytic activity in four of five samples as well as soluble PAD2 content in five of five synovial fluid samples from RA patients. Thus, citrullination apparently can take place extracellularly within inflamed joints, contributing to an enhanced local inflammatory response in ACPA-positive RA patients.

## Abbreviations

ACPA: Anti-citrullinated protein antibody
cFib: Citrullinated fibrinogen
DTT: Dithiothreitol
EDTA: Ethylenediaminetetraacetic acid
ELISA: Enzyme-linked immunosorbent assay
Fib: Fibrinogen
HRP: Horseradish peroxidase
LOD: Limit of detection
mAb: Monoclonal antibody
MQ: ModiQuest
OA: Osteoarthritis
OD: Optical density
PAD: Peptidylarginine deiminase
PBS: Phosphate-buffered saline
RA: Rheumatoid arthritis
rhPAD: Recombinant human peptidylarginine deiminase
SD: Standard deviation
